# Geographic variation of individual venom profile of *Crotalus durissus* snakes

**DOI:** 10.1590/1678-9199-JVATITD-2020-0016

**Published:** 2020-08-26

**Authors:** Leandro Norberto da Silva-Júnior, Lara de Souza Abreu, Caroline Fabri Bittencourt Rodrigues, Nathália da Costa Galizio, Weslei da Silva Aguiar, Caroline Serino-Silva, Valdomiro Souza dos Santos, Isabella Alves Costa, Luis Vicente Franco Oliveira, Sávio Stefanini Sant’Anna, Kathleen Fernandes Grego, Anita Mitico Tanaka-Azevedo, Leandro Nascimento da Silva Rodrigues, Karen de Morais-Zani

**Affiliations:** 1 Laboratory of Herpetology, Butantan Institute, São Paulo, SP, Brazil.; 2 Graduate Program in Human Movement and Rehabilitation (PPGMHR), University Center of Anápolis (UniEVANGÉLICA), Anápolis, GO, Brazil.; 3 Interinstitutional Graduate Program in Biotechnology (PPIB-IPT, IBU and USP), University of São Paulo (USP), São Paulo, SP, Brazil.

**Keywords:** Intraspecific venom variability, Geographic venom variability, Crotalus durissus collilineatus, Crotalus durissus terrificus, Snake venom composition and function

## Abstract

**Background:**

South American rattlesnakes are represented in Brazil by a single species, *Crotalus durissus*, which has public health importance due to the severity of its envenomation and to its wide geographical distribution. The species is subdivided into several subspecies, but the current classification is controversial. In Brazil, the venoms of *C. d. terrificus* and *C. d. collilineatus* are used for hyperimmunization of horses for antivenom production, even though the distinction of these two subspecies are mostly by their geographical distribution. In this context, we described a comparative compositional and functional characterization of individual *C. d. collilineatus* and *C. d. terrificus* venoms from three Brazilian states.

**Methods:**

We compared the compositional patterns of *C. d. terrificus* and *C. d. collilineatus* individual venoms by 1-DE and RP-HPLC. For functional analyzes, the enzymatic activities of PLA_2_, LAAO, and coagulant activity were evaluated. Finally, the immunorecognition of venom toxins by the crotalic antivenom produced at Butantan Institute was evaluated using Western blotting.

**Results:**

The protein profile of individual venoms from *C. d. collilineatus* and *C. d. terrificus* showed a comparable overall composition, despite some intraspecific variation, especially regarding crotamine and LAAO. Interestingly, HPLC analysis showed a geographic pattern concerning PLA_2_. In addition, a remarkable intraspecific variation was also observed in PLA_2_, LAAO and coagulant activities. The immunorecognition pattern of individual venoms from *C. d. collilineatus* and *C. d. terrificus* by crotalic antivenom produced at Butantan Institute was similar.

**Conclusions:**

The results highlighted the individual variability among the venoms of *C. durissus* ssp. specimens. Importantly, our data point to a geographical variation of *C. durissus* ssp. venom profile, regardless of the subspecies, as evidenced by PLA_2_ isoforms complexity, which may explain the increase in venom neurotoxicity from Northeastern through Southern Brazil reported for the species.

## Background

South American rattlesnakes are represented in Brazil by the unique species *Crotalus durissus*, which is distributed throughout a vast portion of the country [1]. The species is subdivided into several subspecies, but the current classification is controversial and, in some cases, poorly supported by molecular characters [2]. Indeed, the assemblage of forms currently known as subspecies of *C. durissus* constitute a set of closely related parapatric forms and the delimitation of subspecies within the *Crotalus durissus* complex has been a lengthy process that is still ongoing and remains largely elusive [3,4].

An analysis of random amplified polymorphic DNA conducted by Echeverrigaray et al. [5] supported the separation of*C. durissus* into*C. d. terrificus*and*C. d. collilineatus*. Conversely, Wuster et al. [2], based on mtDNA data, showed that South American populations of *C. durissus* complex are phylogenetically closely related, and the poorly defined phylogeographical pattern observed south of the Amazon suggests that subspecific distinctions are unwarranted. Thus, the authors consider the subspecies *C. d. cascavella* and *C. d. collilineatus* to be synonymous with *C. d. terrificus*.

In Brazil, *Crotalus durissus* ssp. are of public health importance due to the severity of their envenomation and to their wide geographical distribution [6]. Indeed, they are responsible for the most lethal snakebites events in this country [7]. According to the records of the Brazilian Ministry of Health, although the incidence of crotalic accidents is considered low when compared to the number of envenomations attributed to *Bothrops*sp. (~2,484 and ~20,093 cases, respectively, in 2017), about 0.7% of the envenomation cases caused by *C. durissus* ssp. results in death, against 0.3% of bothropic accidents [7]. 

Currently, immunotherapy with antivenoms is the only available and effective treatment for snakebites. In Brazil, the crotalic F(ab')2 antivenom produced by Butantan Institute (São Paulo, Brazil) is obtained by hyperimmunization of horses with a pool of two *C. durissus* subspecies, *C. d. collilineatus* (50%) and *C. d. terrificus* (50%), nomenclature still adopted despite evidences that these two representatives belong to the same subspecies, as stated above [8].

**Figure 1.**
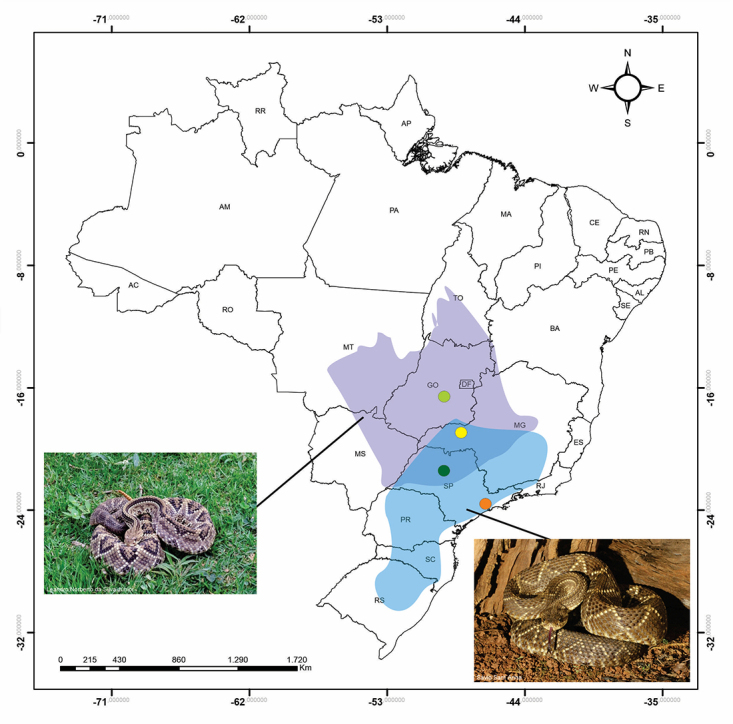
Geographic distribution of *C. d. collilineatus* (purple) and *C. d. terrificus* (blue) and geographic origin of the specimens used throughout this study. The dots indicate the geographic origin of the specimens selected for this work. Light green dot: *C. d. collilineatus* from Goiás state; dark green dot: *C. d. collilineatus* from São Paulo state; yellow dot: *C. d. terrificus* from Minas Gerais state; orange dot: *C. d. terrificus* from São Paulo state.

These two subspecies are distinguished from each other mostly by their geographical distribution and morphological traits, such as the pattern of longitudinal bands on the neck [5,9,10]. *C. d. collilineatus* occurs in central and northeastern Brazil, including parts of Rondônia, Mato Grosso, Goiás, southwestern Bahia, western Minas Gerais, São Paulo (where it intergrades with *C. d. terrificus*) and probably extending southward Paraná [1]. *C. d. terrificus*, in turn, is found in southeastern Brazil, from Rio Grande do Sul and Mato Grosso do Sul north to Minas Gerais [1] (Figure 1). Because the two subspecies are sympatric in São Paulo state [1,11], the classification of specimens collected in this region cannot be achieved unambiguously, due to the possibility of crosses between the two subspecies and the gene flow between them [5].

Due to the wide distribution of the species, *C. durissus* ssp. populations may exhibit a considerable amount of geographic variation [1]. Indeed, a comparative proteomic study showed that the overall composition of*C. d. collilineatus*and *C. d. terrificus* venom are closely related, pointing to geographical variations of the same species, from a venomics perspective [4].

In light of this, and considering the controversial delimitation of subspecies within the *Crotalus durissus* complex [2,4,5,12] as well as the importance of analyzing individual samples in studies involving intraspecific venom variability, we described a comparative compositional and functional characterization of individual *C. d. collilineatus*and *C. d. terrificus* venoms from four geographically distinct regions of three Brazilian states: São Paulo (where the two subspecies intergrades), Minas Gerais and Goiás.

## Methods

### Snakes and venoms

*Crotalus durissus collilineatus*

We selected ten specimens of *C. d. collilineatus* snakes (five males and five females) from São Paulo state (Southeast region of Brazil) (named *C. d. collilineatus* SP) kept in captivity at the Laboratory of Herpetology, Butantan Institute (São Paulo, Brazil). We also selected ten specimens (five males and five females) from Goiás state (Midwest region of Brazil) (named *C. d. collilineatus* GO) kept in captivity at the UniEvangélica University Center of Anápolis (Goiás, Brazil). 

*Crotalus durissus terrificus*

We selected eight specimens of *C. d. terrificus* snakes (four males and four females) from São Paulo state (Southeast region of Brazil) (named *C. d. terrificus* SP) kept in captivity at the Laboratory of Herpetology, Butantan Institute (São Paulo, Brazil). We have also selected seven specimens (four males and three females) from Minas Gerais state (Southeast region of Brazil) (named *C. d. terrificus* MG) also kept in captivity at the Laboratory of Herpetology, Butantan Institute (São Paulo, Brazil). 

The specimens *C. d. collilineatus* and *C. d. terrificus* collected in São Paulo were distinguished from each other by their morphological traits, such as the pattern of longitudinal bands on the neck [5,9,10]. In addition, only adult individuals, with snout-vent length ≥ 80 cm, were selected for this work [13].

These snakes are fed on rodents (*Mus musculus* and/or *Rattus novergicus*) once a month, and their venom is collected sporadically. Information regarding geographic origin of the snakes selected for this work is shown in Figure 1. 

After individual venom extraction, samples were centrifuged for 15 min at 1,700 x g at 4 ºC to remove mucus and cellular debris, lyophilized and stored at -20 ºC until use.

### Protein quantification

Protein concentration was assayed on individual venoms according to the method described by Bradford [14], using the Bio-Rad Protein Assay reagent and bovine serum albumin (BSA) as standard. All samples were assayed in triplicate.

### One-dimensional gel electrophoresis (1-DE)

Twenty micrograms of venom samples were homogenized with sample buffer in the presence or absence of 2-mercaptoethanol. One-DE was carried out in 15% gels [15], and then gels were stained with Coomassie G250 according to manufacturer’s recommendations (GE Healthcare).

### Reversed-phase high performance liquid chromatography (RP-HPLC)

One milligram of lyophilized individual and pooled venoms were dissolved in 1 mL of 0.1% trifluoroacetic acid (TFA; solution A) and centrifuged at 13,000 x g for 15 minutes. Then, 25 μg of venom proteins were separated by RP-HPLC using a Teknokroma Europa Protein 300 C18 column (0.46 cm x 25 cm, 5 mm particle size, 300 Å pore size) and a HPLC system (Jasco). Elution was carried out at 1 mL/min by applying a gradient towards solution B (95% acetonitrile containing 0.1% TFA), according to Gay et al. [16] with some modifications: 5% B for 2.5 min, 5-25% B for 5 min, 25-45% B for 30 min, 45-70% B for 5 min, 70-100% B for 5 min, and 100% B for 5 min. The relative areas of chromatographic peaks (%) were estimated by the software ChromNAV ver.2 (Jasco).

### Phospholipase A_2_ activity

PLA_2_ activity was determined based on the method described by Holzer and Mackessy [17]. Twenty micrograms of venom dissolved in 0.9% saline (in a total volume of 40 μL) were mixed with 200 μL of 10 mM Tris-HCl, 10 mM CaCl_2_, 0.1M NaCl, pH 8.0 in a 96 well microplate. Then, 20 μL of the monodisperse synthetic substrate 4-nitro-3-octanoyloxy-benzoic acid (4-NOBA) (4.16 mM in acetonitrile) was added, to a final concentration of 0.32 mM. After incubation for 60 min at 37 ºC, absorbance values were measured at 425 nm in a microplate reader (SpectraMax i3, Molecular Devices). It was assumed that a change in absorbance of 0.01 is equivalent to 25.8 nM of chromophore release [17]. One unit of PLA_2_ activity corresponds to 1 nM of released chromophore and specific activity was expressed as U/min/mg of venom. All samples were assayed in triplicates. Data were expressed as mean ± SDM.

### L-amino acid oxidase activity

LAAO activity was determined according to Kishimoto and Takahashi [18]. Ten microliters of venom (1 mg/mL) were added to 90 μL of the reaction mixture composed by 250 mM L-Methionine, 2 mM *o*-phenylenediamine (OPD) and 0.8 U/mL horseradish peroxidase, in 50 mM Tris pH 8.0 buffer. After incubation at 37 °C for 30 min, the reaction was stopped with the addition of 50 μL of 2 M H_2_SO_4_, and absorbances were measured at 492 nm in a SpectraMax i3 microplate reader (Molecular Devices). LAAO activity was indirectly estimated using a standard curve of H_2_O_2_. One U of LAAO activity corresponds to 1 mM of H_2_O_2_ produced and specific activity was expressed as U/min/mg of venom. All samples were assayed in triplicates. Data were expressed as mean ± SDM.

### Coagulant activity

*Coagulant activity upon human plasma*

Coagulant activity of venom was determined in samples of human citrated plasma. Briefly, 200 µL of human plasma were incubated for 1 min at 37 ºC followed by the addition of 100 µL venom samples (solubilized in saline solution 0.9% for a concentration of 250 µg/mL). Immediately after venom addition, the coagulation time was recorded. All clotting time were measured on a coagulometer (Drake). All samples were assayed in triplicates. Data were expressed as mean ± SDM.

*Thrombin-like activity upon bovine fibrinogen*

Coagulant activity of venom was also determined in samples of 2 mg/mL bovine fibrinogen. Briefly, 200 µL of fibrinogen solution were incubated for 1 min at 37 °C followed by the addition of 100 µL venom samples (solubilized in saline solution 0.9% for a concentration of 250 µg/mL). Immediately after venom addition, the coagulation time was recorded. All clotting time were measured on a coagulometer (Drake). All samples were assayed in triplicates. Data were expressed as mean ± SDM.

*Thrombin-like activity upon chromogenic substrate*

The chromogenic substrate S-2238 (Chromogenix) were used to assess the thrombin-like activity of the serine proteases according to the manufacturer's recommendations, with some modifications. Five microliters of 1 mg/mL venom (resuspended in 0.9% saline) were incubated with 10 μL of chromogenic substrate S-2238 (4 mM) and 90 μL of 50 mM Tris pH 8.0 at 37 °C for 5 min. The reaction was stopped by the addition of 90 μL of 20% acetic acid and the absorbance values were measured at 405 nm in a microplate reader (SpectraMax i3, Molecular Devices). Bovine thrombin (2 U/mL) (Roche) was used as positive control. We defined a change in absorbance of 0.009 as corresponding to 1 U thrombin-like activity and specific activity was expressed as U/min/mg of venom. All samples were assayed in triplicates. Data were expressed as mean ± SDM.

### Western blotting

The crotalic polyvalent F(ab')_2_ antivenom (*soro anticrotálico* - SAC) used in this immunorecognition assay was provided by Butantan Institute (São Paulo, Brazil), and was produced by hyperimmunization of horses using a pool of two *Crotalus durissus* subspecies, namely *C. d. terrificus* (50%) and *C. d. collilineatus* (50%). Venom samples (20 µg) separated by 15% SDS-PAGE were electrotransferred at 15 V for 35 min onto PVDF membranes. The membranes were blocked with TBS-milk overnight at 4 °C. The membrane was incubated with 1:1,000 SAC for 2 h at room temperature. After washing the blots with Tris-HCl buffer (10 mM Tris, 150 mM NaCl, pH 7.5) containing 0.1% Tween 20, the membranes were exposed to 1:10,000 peroxidase-labelled anti-horse IgG (Sigma) for 2 h at room temperature. After washing off unbound secondary antibodies, the immunoreactive bands were visualized using diaminobenzidine (Sigma) and H_2_O_2_.

### Statistical analyses

Results were statistically analyzed using one-way analysis of variance (ANOVA), followed by Bonferroni test. Differences with p < 0.05 were considered statistically significant. Statistical analyses were performed using GraphPad Prism software (version 8).

### Animal ethics statement

All procedures involving the use of animals were performed in accordance with the Guide for the Care and Use of Laboratory Animals (1996) and were approved by the Ethical Committee for the Use of Animals of Butantan Institute (protocol number 7803090818) and UniEvangélica (004/2019).

## Results and discussion

### Compositional analysis

**Figure 2.**
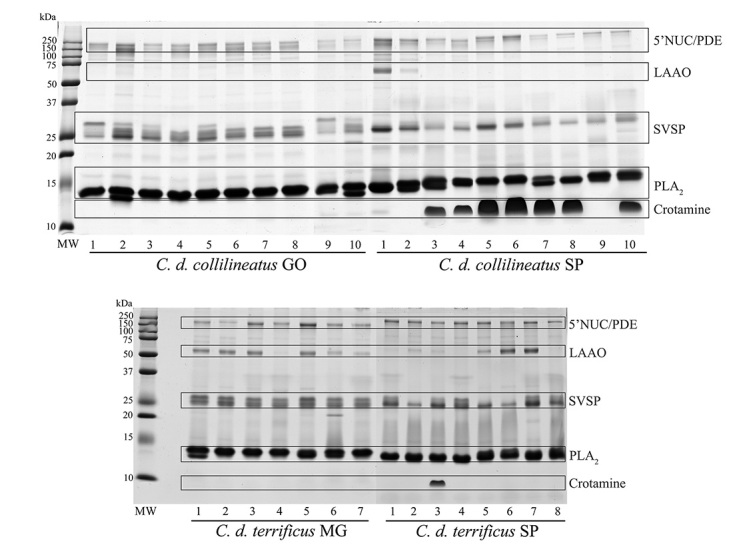
Electrophoretic profile of *C. d. collilineatus* and *C. d. terrificus* venom. Individual venom samples (20 µg) are subjected to SDS-PAGE 15%, under non reducing conditions, and proteins were stained using Coomassie G (GE Healthcare). *C. d. collilineatus* GO: specimens from Goiás state; *C. d. collilineatus* SP: specimens from São Paulo state; *C. d. terrificus* MG: specimens from Minas Gerais state; *C. d. terrificus* SP: specimens from São Paulo state; MW: molecular weight marker (Dual Color Precision Plus Protein Standards - BioRad).

**Figure 3.**
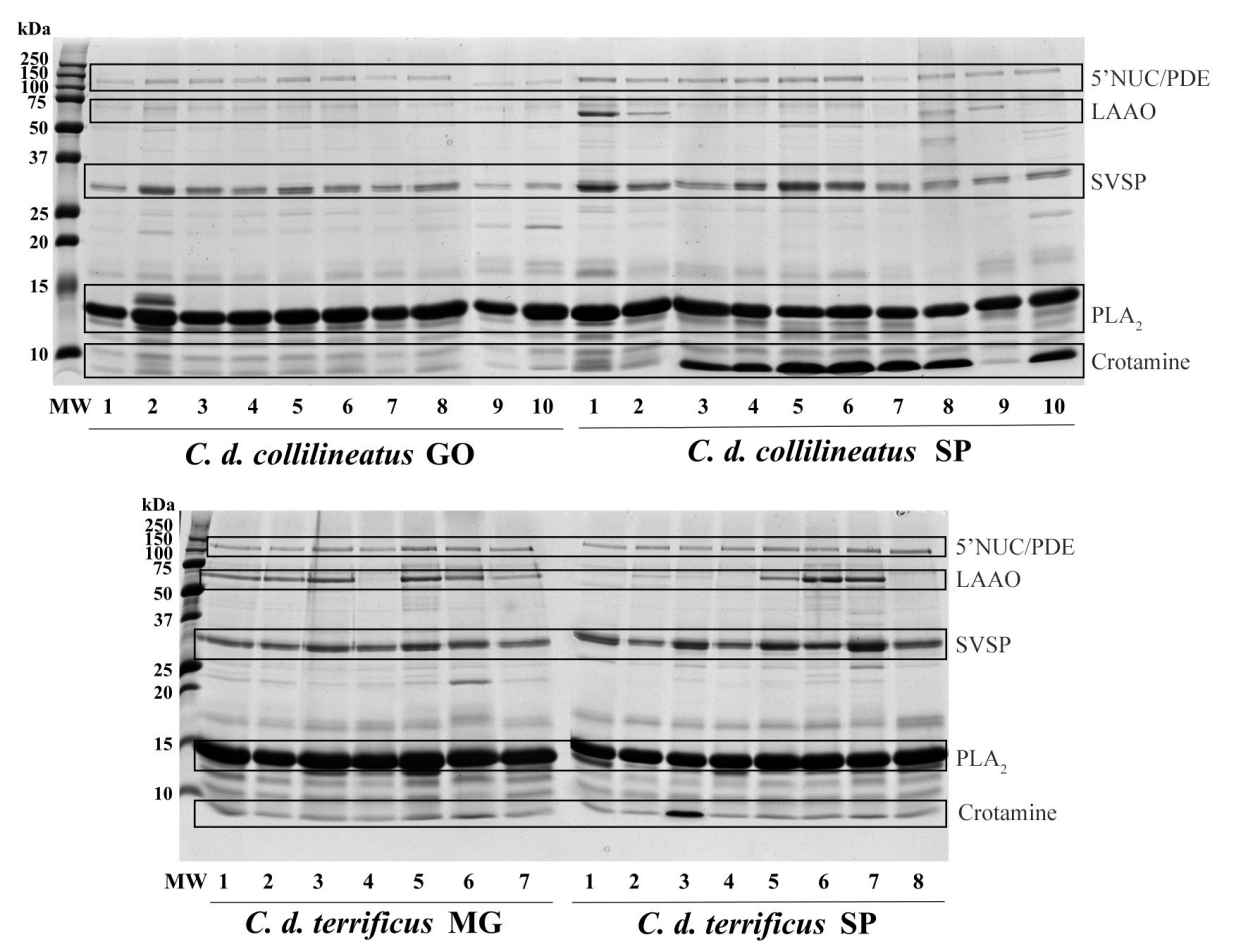
Electrophoretic profile of *C. d. collilineatus* and *C. d. terrificus* venom. Individual venom samples (20 µg) are subjected to SDS-PAGE 15%, under reducing conditions, and proteins were stained using Coomassie G (GE Healthcare). *C. d. collilineatus* GO: specimens from Goiás state; *C. d. collilineatus* SP: specimens from São Paulo state; *C. d. terrificus* MG: specimens from Minas Gerais state; *C. d. terrificus* SP: specimens from São Paulo state; MW: molecular weight marker (Dual Color Precision Plus Protein Standards - BioRad). The main protein bands were assigned to its major components. 5’NUC: 5’nucleotidases; PDE: phosphodiesterases; LAAO: L-amino acid oxidases; SVSP: snake venom serine proteases, PLA_2_: phospholipases A_2_.

The protein profile of individual venoms from *C. d. collilineatus* and *C. d. terrificus* snakes was first analyzed by 1-DE, revealing a comparable overall band composition, in non-reducing and reducing conditions (Figures 2 and 3, respectively). Considering that the electrophoretic profile and the toxin composition of *C. durissus* ssp. venom have already been deeply characterized [4,12,19-22], protein bands were assigned, based on their molecular masses, to the main protein families that compose *C. durissus* ssp. venom [4]. 

As expected, a band with ~14 kDa in non-reducing and reducing conditions, corresponding to PLA_2_s, was observe in all venom samples. Crotoxin, a β-neurotoxin that inhibits the release of acetylcholine at the neuromuscular junction [23,24], is the main component of *C. durissus* ssp. venom. This toxin is a heterodimeric complex linked by non-covalent bonds, composed by a basic PLA_2_ (chain B, ~14 kDa) with neurotoxic and enzymatic activity, and crotapotin (chain A, with ~9 kDa), a non-toxic acidic protein that increases the lethal potential of the neurotoxic PLA_2_ [23,25-28]. 

In addition, the protein bands generally assigned to the thrombin-like enzyme gyroxin (a serine proteinase with ~30 kDa, in both non reducing and reducing conditions) and to the C-type lectin-like convulxin (~100 and 10-14 kDa, in non-reducing and reducing conditions, respectively) are present in all venom samples, despite differences in band intensities. 

Not surprisingly, we observed a remarkable intraspecific variability regarding the presence of crotamine. This ~10 kDa myotoxin is present only in the venom of specimens from São Paulo state, despite the subspecies (seven out ten *C. d. collilineatus* and only a single individual of *C. d terrificus*). Indeed, Boldrini-França et al. [4] have demonstrated that there is an increase in the relative abundance of crotamine among *C. durissus* ssp. coincident with the direction of the dispersal of this taxa, in the north-south direction of the South American continent, across a central Amazonian corridor during the middle Pleistocene [29].

Another protein band displaying high qualitative and quantitative variability among species is L-amino acid oxidase (LAAO) (~58 kDa, under non-reducing and reducing conditions). This band was visually identified in individual venoms of four *C. d. collilineatus* SP, six *C. d. terrificus* MG and three *C. d. terrificus* SP. The presence of this enzyme is commonly associated with yellow color in snake venoms [30], and although this toxin family has been extensively researched due to its pharmacological and biotechnological potential (for review, please refer to [31,32]), its functional role in subduing prey and it effect on human envenomation are not fully elucidated.

Intraspecific variation regarding the presence/absence of the putative LAAO band and the associated venom color has been previously documented in *C. d. collilineatus* venom [20]. However, the driving mechanisms that lead to such variability are unknown.

**Figure 4.**
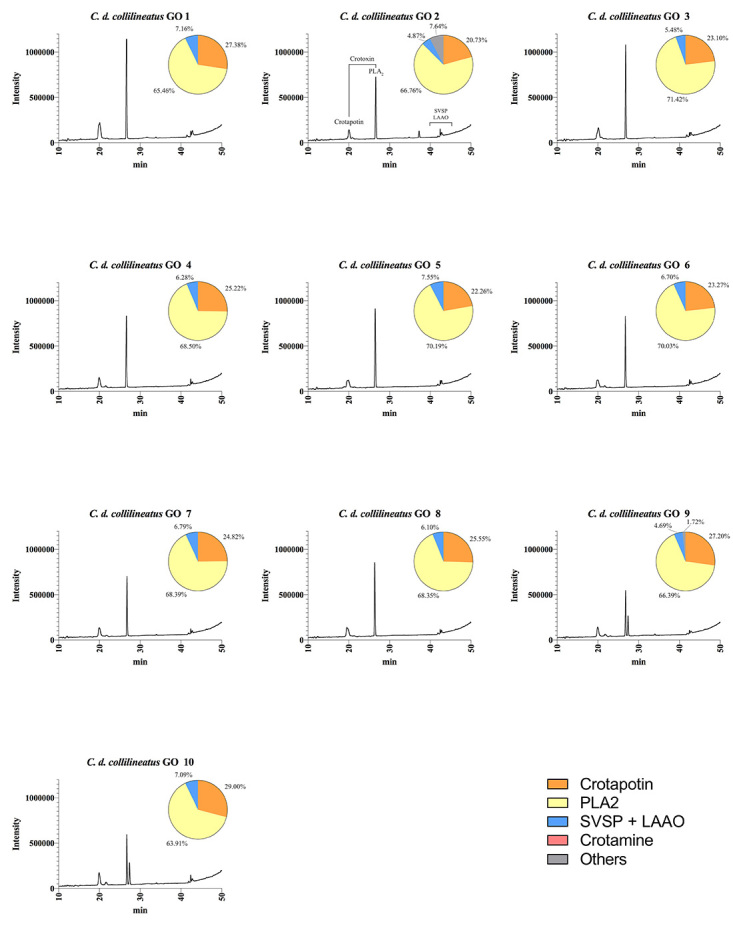
Elution profiles of individual *C. d. collilineatus* venom from Goiás state by RP-HPLC. Samples of 25 μg of lyophilized venom were dissolved in 0.1% trifluoroacetic acid (TFA) and 5% acetonitrile (solution A) and subjected to RP-HPLC on a C18 column. Elution was performed at 1.0 mL/min by applying a gradient toward 0.1% TFA and 95% acetonitrile (solution B), as described in the experimental section. The main chromatographic peaks of one representative were assigned to its major components. This representative was selected based on the presence/absence and intensity of particular chromatographic peaks. LAAO: L-amino acid oxidases; SVSP: snake venom serine proteases, PLA_2_: phospholipases A_2_.

**Figure 5.**
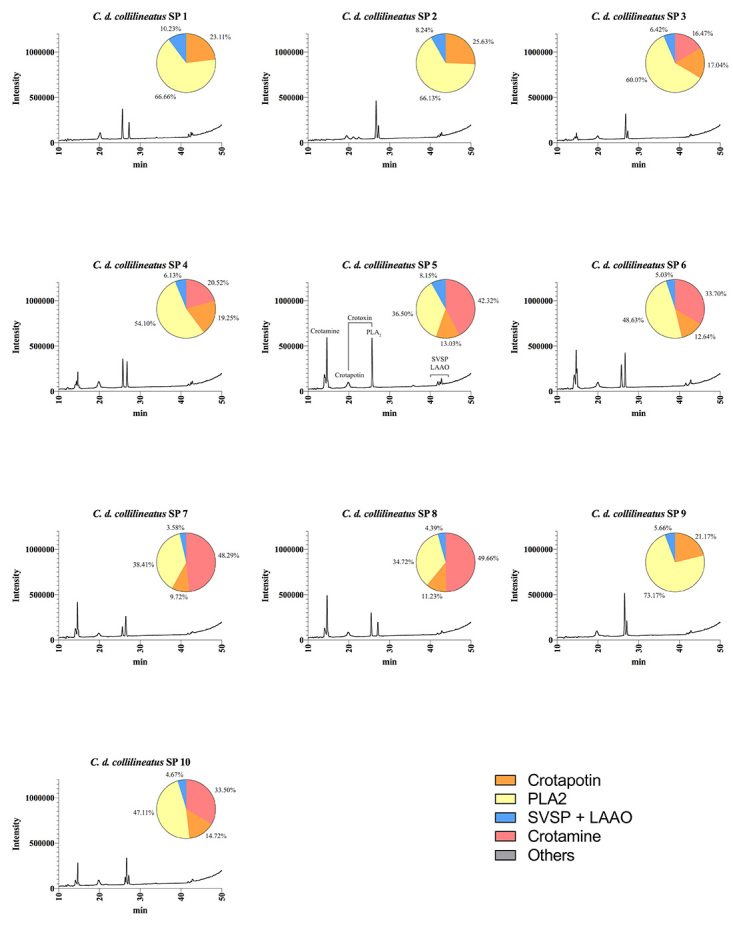
Elution profiles of individual *C. d. collilineatus* venom from São Paulo state by RP-HPLC. Samples of 25 μg of lyophilized venom were dissolved in 0.1% trifluoroacetic acid (TFA) and 5% acetonitrile (solution A) and subjected to RP-HPLC on a C18 column. Elution was performed at 1.0 mL/min by applying a gradient toward 0.1% TFA and 95% acetonitrile (solution B), as described in the experimental section. The main chromatographic peaks of one representative were assigned to its major components. This representative was selected based on the presence/absence and intensity of particular chromatographic peaks. LAAO: L-amino acid oxidases; SVSP: snake venom serine proteases, PLA_2_: phospholipases A_2_.

**Figure 6.**
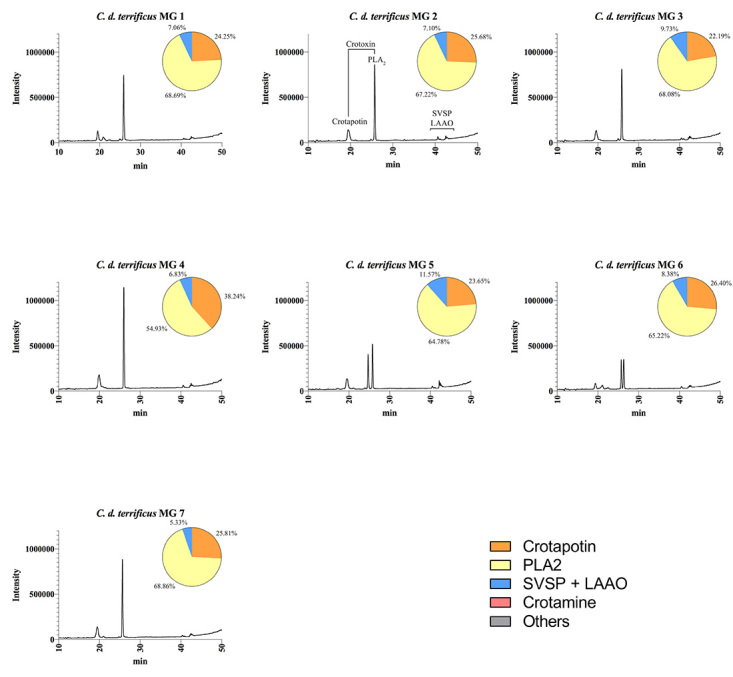
Elution profiles of individual *C. d. terrificus* venom from Minas Gerais state by RP-HPLC. Samples of 25 μg of lyophilized venom were dissolved in 0.1% trifluoroacetic acid (TFA) and 5% acetonitrile (solution A) and subjected to RP-HPLC on a C18 column. Elution was performed at 1.0 mL/min by applying a gradient toward 0.1% TFA and 95% acetonitrile (solution B), as described in the experimental section. The main chromatographic peaks of one representative were assigned to its major components. This representative was selected based on the presence/absence and intensity of particular chromatographic peaks. LAAO: L-amino acid oxidases; SVSP: snake venom serine proteases, PLA_2_: phospholipases A_2_.

**Figure 7.**
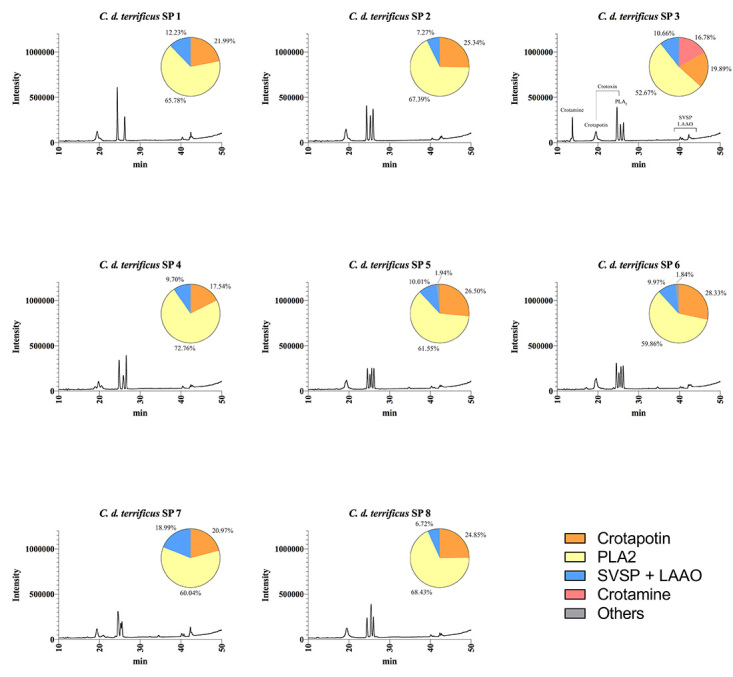
Elution profiles of individual *C. d. terrificus* venom from São Paulo state by RP-HPLC. Samples of 25 μg of lyophilized venom were dissolved in 0.1% trifluoroacetic acid (TFA) and 5% acetonitrile (solution A) and subjected to RP-HPLC on a C18 column. Elution was performed at 1.0 mL/min by applying a gradient toward 0.1% TFA and 95% acetonitrile (solution B), as described in the experimental section. The main chromatographic peaks of one representative were assigned to its major components. This representative was selected based on the presence/absence and intensity of particular chromatographic peaks. LAAO: L-amino acid oxidases; SVSP: snake venom serine proteases, PLA_2_: phospholipases A_2_.

We further characterized the protein profile of individual venoms by RP-HPLC, which highlighted the intraspecific variability of *C. durissus* ssp. venom regarding its two major components, crotoxin (chains A and B) and crotamine (Figures 4, 5, 6 and 7). The HPLC venom profile of *C. durissus* ssp. has been well characterized by several authors [4,20-22] and, based on these previous reports, the main chromatographic peaks were assigned to its major components.

RP-HPLC analysis corroborated SDS-PAGE results concerning the presence/absence of crotamine, eluted at ~15 min using the methodology described herein. The results highlight the qualitative and quantitative variation of crotamine, whose corresponding chromatographic peak is present only in the venom of *C. d. collilineatus* and *C. d. terrificus* individuals from São Paulo state (Figures 5 and 7, respectively).

When submitted to RP-HPLC, the two sub-units of crotoxin are separated: crotapotin (chain A) is eluted first (at 20 min), followed by PLA_2_ (chain B) (eluted at 25-30 min). Interestingly, most individual venom samples from *C. d. collilineatu*s GO and from *C. d. terrificus* MG showed only a single peak assigned to PLA_2_ (eight out of ten *C. d. collilineatu*s GO and five out seven *C. d. terrificus* MG) (Figures 4 and 6). Conversely, most individual venoms from São Paulo state, despite the subspecies, showed two or more chromatographic peaks associated to this toxin family (nine out of ten *C. d. collilineatu*s SP and all *C. d. terrificus* SP) (Figures 5 and 7). In this regard, venom from *C. d. terrificus* SP showed a higher variability, in terms of number of chromatographic peaks, compared to *C. d. collilineatu*s SP, presenting two to four peaks corresponding to PLA_2_s. This observation points to a higher complexity of PLA_2_ isoforms in *C. durissus* ssp. venom from São Paulo state. Several crotoxin isoforms have been described [33,34], which vary in their biological activity, probably as a result of the heterogeneity in PLA_2_and crotapotin isoforms [33]. Interestingly, Boldrini-França et al. [4] reported an evolutionary trend toward increasing neurotoxicity to mice among *C. durissus* ssp. from Northeastern through Southern Brazil, along the dispersal route of this taxa. In this sense, it is tempting to relate this trend to the higher complexity regarding PLA_2_ isoforms observed in individual HPLC venom profiles reported herein. However, the compositional analyses of individual venom samples from *C. durissus* ssp. from the south region of Brazil would be important to elucidate if there is a geographical trend of increasing complexity of PLA_2_ isoforms. 

The relative abundance of each peak in HPLC venom profile was estimated and although highly variable values were obtained for the main toxin families, this variability could not be assigned to a specific subspecies (Figures 4, 5, 6 and 7). The main difference is related to the content of crotamine, identified only in specimens from São Paulo, which varies from 16.47 to 49.99% according to the software used. On the other hand, the relative abundance of the chromatographic peaks assigned to PLA_2_s varied from 35 to 70%, regardless of the number of isoforms identified in HPLC profile. However, due to the high content of crotamine estimated in some *C. d. collilineatus* SP venoms (> 30%), the relative abundance of PLA_2_s in these crotamine-positive individuals is lower (< 50%) when compared to the other specimens analyzed.

It is important to point out that the assignment of protein bands and chromatographic peaks to toxin families based on their molecular masses and retention times, respectively, is elusive and deserves further investigation. Furthermore, the determination of the relative abundance of the main toxin families by a combination of HPLC, SDS-PAGE and mass spectrometry (“venomics” approach [35]) would provide more accurate results. Nevertheless, the comparison of individual venom protein profiles by SDS-PAGE and RP-HPLC gives information regarding intraspecific variability related to the subspecies or the geographic origin of the specimens.

### Enzymatic activities

Catalytic activities of the main classes of enzymes composing *C. durissus* ssp. venom corroborated the individual differences predicted by our compositional results. 

**Figure 8.**
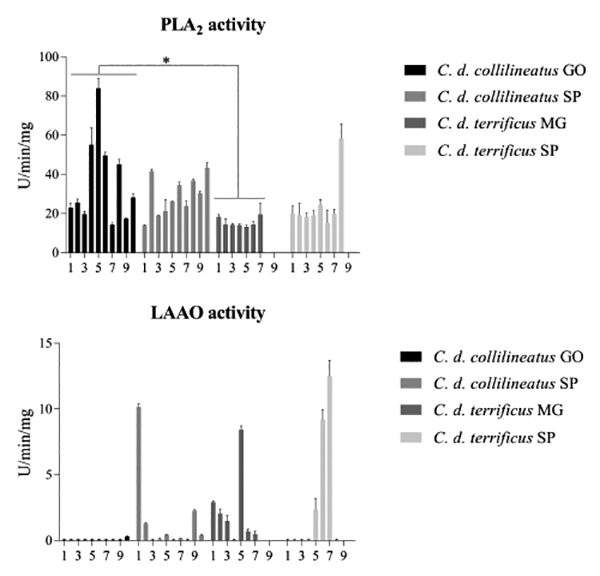
Enzymatic activities of individual *C. d. collilineatus* and *C. d. terrificus* venom. (**A**) Phospholipase A_2_ activity and (**B**) L-amino acid oxidase activity. Results were expressed as mean ± SDM. *C. d. collilineatus* GO: specimens from Goiás state; *C. d. collilineatus* SP: specimens from São Paulo state; *C. d. terrificus* MG: specimens from Minas Gerais state; *C. d. terrificus* SP: specimens from São Paulo state; *p < 0.05.

Concerning PLA_2_ enzymatic activity, such variability is more prominent in *C. d. collilineatus* venom, despite the geographic origin of the specimens (Figure 8A). In *C. d. terrificus* venom, this activity is more homogeneous, except for individual 7, which showed higher hydrolytic activity upon the synthetic substrate NOBA. At a first glance, PLA_2_ activity seems to be higher in *C. d. collilineatus*, as reported previously [12]; however, significant differences (p < 0.05) were identified only between venoms from *C. d. collilineatus* GO and *C. d. terrificus* MG. 

In addition, a remarkable qualitative and quantitative individual variability was noticed among individual venom samples regarding LAAO activity (Figure 8B). Enzymatic LAAO activity was identified in 1 *C. d. collilineatus* GO, 5 *C. d. collilineatus* SP, 6*C. d. terrificus* MG and 3 *C. d. terrificus* SP individual venoms, and, in general, correlates with the presence and the intensity of the protein band with ~58 kDa, as discussed in the previous section. Although it is not possible to visually identify this particular protein band by SDS-PAGE (Figure 2) in the venom of specimen 10 of *C. d. collilineatus* GO and specimen 5 of *C. d. collilineatus* SP, they displayed a negligible LAAO activity. Moreover, LAAO enzymatic activity also correlates with yellow venom color. All *C. d. collilineatus* GO venom samples are white and only three *C. d. collilineatus* SP are yellow (samples 1, 2 and 9). In addition, six individual venoms of *C. d. terrificus* MG (1, 2, 3, 5, 6 and 7) and three venom samples of *C. d. terrificus* SP are yellow (5, 6 and 7). The high intraspecific variability regarding LAAO activity was expected based on the compositional characterization of *C. durissus* ssp. described herein and in previous reports [20,21].

The pro-coagulant activity exerted by *C. durissus* ssp. venom is mainly due to the action of the serine proteinase gyroxin [36,37], although C-type lectins and metalloproteinases, even in low amounts as those reported in the venom of this species [4,21,37], may also be involved. 

Gyroxin promotes unusual breakage of fibrinogen to fibrinopeptide A, resulting in a soluble form of fibrin that is more susceptible to the action of fibrinolytic agents [36,38-40]. The action of this thrombin-like enzyme may result in a complete lack of blood clotting in severe envenomation cases caused *C. durissus* ssp. due to fibrinogen consumption [41].

**Figure 9.**
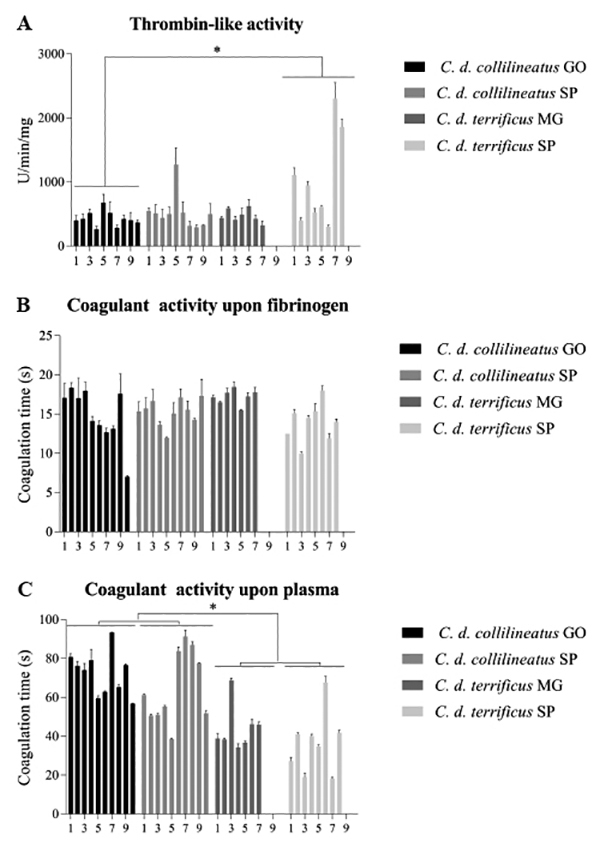
Coagulant activity of individual *C. d. collilineatus* and *C. d. terrificus* venom. (**A**) Thrombin-like activity upon the chromogenic substrate S-2238 (Chromogenix), (**B**) thrombin-like activity upon bovine fibrinogen, and (**C**) coagulant activity upon human plasma. Results were expressed as mean ± SDM. *C. d. collilineatus* GO: specimens from Goiás state; *C. d. collilineatus* SP: specimens from São Paulo state; *C. d. terrificus* MG: specimens from Minas Gerais state; *C. d. terrificus* SP: specimens from São Paulo state; *p < 0.05.

The thrombin-like activity of individual venoms of *C. d. collilineatus* and *C. d. terrificus* was assessed using the chromogenic substrate S-2238 and bovine fibrinogen (Figure 9A and 9B). Thrombin-like activity upon the chromogenic substrate does not agree with the results obtained using bovine fibrinogen as substrate. For example, the specimen 7 of *C. d. terrificus* SP displayed the higher activity upon S-2238 amongst all samples analyzed, while its activity upon bovine fibrinogen was lower than most of the venoms. Indeed, differences in substrate specificity have already been reported for the thrombin-like activity of *C. durissus* ssp. venom, which is higher upon human fibrinogen when compared to its activity on bovine, rabbit and rat fibrinogen [12].

Despite the individual variations in thrombin-like activity of individual venoms, a significant difference regarding this activity was observed between groups *C. d. collilineatus* GO and *C. d. terrificus* SP (p < 0.05), probably due to the high activity of some *C. d. terrificus* SP individuals venom (specially individuals 7 and 8) upon the chromogenic substrate S-2238 (Figure 9A). However, the comparison among the other experimental groups showed no substantial differences.

Venoms from *C. d. terrificus* showed significantly more coagulant activity on human plasma than *C. d. collilineatus* venoms (p < 0.05) (Figure 9C), independently of their geographical origin. The results of the coagulant activity of individual venoms on human plasma do not match their thrombin-like activity. Besides substrate specificity, this observation indicates that different toxins (and their synergistic action) and different plasmatic targets may be involved in the coagulation disturbances caused by *C. durissus*ssp envenomation.

### Immunorecognition analysis

As stated previously in this work, crotalic antivenom is produced using a mixture of venoms from *C. d. terrificus* and *C. d. collilineatus*, using the same proportion of each one. However, the venom pool used as antigen is composed mainly (but not exclusively) by venoms from specimens from the southeastern Brazilian states. Although only slight compositional and functional differences were noticed between *C. d. terrificus* and *C. d. collilineatus* venoms from Goiás, Minas Gerais and São Paulo states, it was important to evaluate whether these differences could affect the immunorecognition of venom proteins by crotalic polyvalent F(ab’)_2_ antivenom produced by Butantan Institute. 

**Figure 10.**
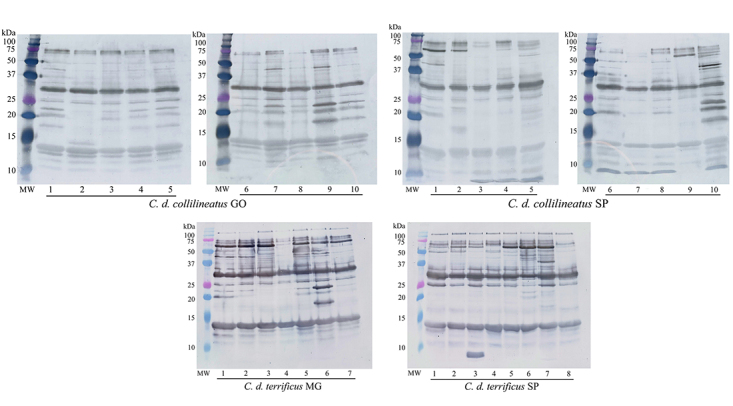
Immunorecognition profile obtained of individual *C. d. collilineatus* and *C. d. terrificus* venom by anticrotalic antivenom produced by Butantan Institute by Western blotting. Venom proteins (20 μg) were subjected to SDS-PAGE 15% under reducing conditions and electrotransferred to a PVDF membrane. Membrane was sequentially incubated with anti-bothropic antivenom and peroxidase-conjugated anti-horse IgG. The reaction was developed using DAB and H_2_O_2_. MW: molecular weight marker (Dual Color Precision Plus Protein Standards - BioRad); *C. d. collilineatus* GO: specimens from Goiás state; *C. d. collilineatus* SP: specimens from São Paulo state; *C. d. terrificus* MG: specimens from Minas Gerais state; *C. d. terrificus* SP: specimens from São Paulo state.

To this end, individual venoms of *C. d. terrificus* from SP and MG, and individual venoms of *C. d. collilineatus* from SP and GO, were subjected to Western blotting under reducing conditions, showing similar immunorecognition profiles (Figure 10). All the major protein bands, including those associated to PLA_2_ (crotoxin) (~14 kDa) and gyroxin (~30 kDa), were recognized by crotalic antivenom in all individuals. 

Interestingly, the protein band assigned to crotamine (~10 kDa) was easily detected, despite a previous report of a weak immunorecognition of this toxin by the antivenom produced by Butantan Institute [4]. These contradictory observations may be attributed to differences in the composition of crotalic venom pools used to produce each batch of antivenom, regarding crotamine content. 

In addition, the protein band corresponding to LAAO (~58 kDa) also showed recognition in all individuals which presented this enzyme. Our results support those described by Santoro et al (1999), who reported no differences in the immunorecognition pattern of *C. d. collilineatus*, *C. d. ruruima* and *C. d. terrificus* using the same antivenom [12].

The venom composition variability in subspecies of North American rattlesnakes has also been reported. The investigation of the diversity of toxins present in *Crotalus oreganus helleri*, across its geographic range, revealed significant differences in venoms of the four populations analyzed [42]. In addition, HPLC analysis combined to mass spectrometry identification revealed that the protein profile and the relative abundance of protein families in *Sistrurus catenatus catenatus*, *S. c. tergeminus* and *S. c. edwardsii* are not conserved [43]. In contrast, the proteomic analysis of the venoms of *S. miliarius streckeri* and *S. m. miliarius* showed that these venoms exhibit the same general classes of proteins as those found in other *Sistrurus* species but differ in their relative abundances of specific protein families [44], similarly to what was observed in the present work.

The origin of phenotypic variation in snakes’ venoms and its retention in a population are central issues for understanding evolutionary adaptations [45]. In addition, the identification of the processes involved in geographical variability of venom composition and function in snake species and subspecies with a continuous spatial distribution, as *C. d. collilineatus* and *C. d. terrificus*, is a challenging task [46]. Variation in venom composition at different biological levels is widespread and has been attributed to a number of factors, such as phylogenetic affinities, snake’s age, geography, diet and environmental conditions [43,45,47-52]. 

Calvete et al. [50] reported that the venom of South American rattlesnakes has retained juvenile venom characteristics in the adult along their North-South dispersal, and the venom of *C. d. terrificus* and *C. d. collilineatus* display this pattern (paedomorphism). Furthermore, the diet is similar for both subspecies *C. d. terrificus* and *C. d. collilineatus*, which have specialist feeding habit and prey on mammals during their whole lifespan [53]. However, the hypothesis that the specific prey items available across their wide geographical distribution may account for the PLA_2_ variability described herein, should not be discarded and deserves further investigation, since the presence of prey-specific toxins has already been described in some snake venoms [54-57].

It is recognized that future work involving the identification of PLA_2_ isoforms described in the present work as well as toxicity tests is needed to further characterize the geographic variation of *C. durissus* ssp. venom. Nevertheless, taken together, our results represent a significant step toward characterizing the intraspecific venom variability present in this species.

## Conclusion

In this work, we reported the compositional and enzymatic profile of individual venoms from *C. d. collilineatus* and *C. d. terrificus* from different Brazilian regions. We identified remarkable individual variability among the venoms of the specimens of *C. durissus*ssp. selected for this study. Importantly, the results show geographical variation of *C. durissus* ssp. venom profile, regardless of the subspecies, as evidenced by PLA_2_ isoforms complexity, which may explain the increase in venom neurotoxicity reported for the species from Northeastern through Southern Brazil. Although the degree of correlation between snake venom variation and levels of phylogenetic divergence between species is an open question [44], this report supports the findings described by Boldrini-França et al. [4], who suggested that, from a venomic point of view, *C. d. collilineatus* and *C. d. terrificus* may represent geographical variations of the same species.
